# Liraglutide and its Neuroprotective Properties—Focus on Possible Biochemical Mechanisms in Alzheimer’s Disease and Cerebral Ischemic Events

**DOI:** 10.3390/ijms20051050

**Published:** 2019-02-28

**Authors:** Michał Wiciński, Maciej Socha, Bartosz Malinowski, Eryk Wódkiewicz, Maciej Walczak, Karol Górski, Maciej Słupski, Katarzyna Pawlak-Osińska

**Affiliations:** 1 Department of Pharmacology and Therapeutics, Faculty of Medicine, Collegium Medicum in Bydgoszcz, Nicolaus Copernicus University, M. Curie 9, 85-090 Bydgoszcz, Poland; wicinski4@wp.pl (M.W.); bartosz.malin@gmail.com (B.M.); eryk.wodkiewicz09@gmail.com (E.W.); karolgorski-2@gazeta.pl (K.G.); 2 Department of Obstetrics, Gynecology and Gynecological Oncology, Faculty of Medicine, Collegium Medicum in Bydgoszcz, Nicolaus Copernicus University, Ujejskiego 75; 85-168 Bydgoszcz, Poland; msocha@copernicus.gda.pl (M.S.); osinskak1@wp.pl (K.P.-O.); 3 Department of Hepatobiliary and General Surgery. Faculty of Medicine, Collegium Medicum in Bydgoszcz, Nicolaus Copernicus University, M. Curie 9, 85-090 Bydgoszcz, Poland; maciej.slupski@cm.umk.pl; 4 Department of Pathophysiology of Hearing and Balance System, Faculty of Medicine, Collegium Medicum in Bydgoszcz, Nicolaus Copernicus University, M. Curie 9, 85-090 Bydgoszcz, Poland

**Keywords:** liraglutide, neuroprotection, pathways, inflammation, Alzheimer’s disease, stroke

## Abstract

Liraglutide is a GLP-1 analog (glucagon like peptide-1) used primarily in the treatment of diabetes mellitus type 2 (DM2) and obesity. The literature starts to suggest that liraglutide may reduce the effects of ischemic stroke by activating anti-apoptotic pathways, as well as limiting the harmful effects of free radicals. The GLP-1R expression has been reported in the cerebral cortex, especially occipital and frontal lobes, the hypothalamus, and the thalamus. Liraglutide reduced the area of ischemia caused by MCAO (middle cerebral artery occlusion), limited neurological deficits, decreased hyperglycemia caused by stress, and presented anti-apoptotic effects by increasing the expression of Bcl-2 and Bcl-xl proteins and reduction of Bax and Bad protein expression. The pharmaceutical managed to decrease concentrations of proapoptotic factors, such as NF-κB (Nuclear Factor-kappa β), ICAM-1 (Intercellular Adhesion Molecule 1), caspase-3, and reduced the level of TUNEL-positive cells. Liraglutide was able to reduce the level of free radicals by decreasing the level of malondialdehyde (MDA), and increasing the superoxide dismutase level (SOD), glutathione (GSH), and catalase. Liraglutide may affect the neurovascular unit causing its remodeling, which seems to be crucial for recovery after stroke. Liraglutide may stabilize atherosclerotic plaque, as well as counteract its early formation and further development. Liraglutide, through its binding to GLP-1R (glucagon like peptide-1 receptor) and consequent activation of PI3K/MAPK (Phosphoinositide 3-kinase/mitogen associated protein kinase) dependent pathways, may have a positive impact on Aβ (amyloid beta) trafficking and clearance by increasing the presence of Aβ transporters in cerebrospinal fluid. Liraglutide seems to affect tau pathology. It is possible that liraglutide may have some stem cell stimulating properties. The effects may be connected with PKA (phosphorylase kinase A) activation. This paper presents potential mechanisms of liraglutide activity in conditions connected with neuronal damage, with special emphasis on Alzheimer’s disease and cerebral ischemia.

## 1. Introduction

There are many disease states whose occurrence is connected to neural damage. The second most common cause of death in the world is stroke, which kills 6 million people a year. Neurodegenerative diseases and Alzheimer’s disease among them contribute to over 1.5 million deaths each year [1]. The above-mentioned data lead researchers around the world to seek new drugs that may help renew nerve cells or protect them from damage. Recently, there has been a growing number of reports suggesting that GLP-1 analogs (glucagon like peptide-1), with liraglutide being a representative of which, may have neuroprotective effects. Liraglutide is a pharmaceutical used primarily in the treatment of diabetes mellitus type 2 (DM2) and obesity. It binds to GLP-1 receptor and stimulates the secretion of insulin by lowering blood glucose levels in a way that depends on its plasma concentration. As a result, the risk of hypoglycemia is very low [2]. The drug was also reported to delay gastric emptying [3], inhibit glucagon secretion [4], reduce appetite, inhibit weight gain [5], and lower blood triglyceride levels [6]. What is crucial for potential neurological benefits is that it exceeds the blood-brain barrier [7] and is resistant to the action of enzyme metabolizing endogenous GLP-1, called dipeptidyl peptidase 4 (DPP-4). Therefore, the half-life of liraglutide surpasses the duration of its natural analog and reaches about 13 h [8]. GLP-1 agonists can improve insulin sensitivity, and by that make an impact on cellular metabolism as well as affect it directly through GLP-1Rs (glucagon like peptide-1 receptors). When a GLP-1 agonist, such as liraglutide, binds to its receptor, signaling pathways that converge with the insulin-signaling pathway are activated [9]. The process facilitates insulin signaling by downstream modulation of various factors, such as PKA (phosphorylase kinase A), PI3K (Phosphoinositide 3-kinase), MAPK (mitogen associated protein kinase), PKC (Protein kinase C), and AKT (protein kinase B) [10]. Insulin and IGF-1 (insulin-like growth factor-1) show structural homology and close resemblance in terms of biological activity [11,12]. Although they are primarily produced and secreted peripherally by the pancreas and liver, both proteins are also synthesized in the CNS (central nervous system), and through their receptors (IGF-1R and IR) contribute to neuronal outgrowth and survival, synaptic maintenance, as well as to learning and memory [13]. IGF-1R and IR possess tyrosine kinase activity and are able to phosphorylate the intramembrane domains serving as docking sites for insulin receptor substrate (IRS) [14]. IRS plays a key role in transmitting signals from the insulin and (IGF-1) receptors to intracellular pathways. The IRS has multiple potential phosphorylation sites, which implies that insulin/IGF-1 signaling pathway can be regulated by ligand-independent processes [15]. Effects of GLP-1R activation can be subdivided into those leading to acute and chronic response [16]. Acute results like insulin secretion, exocytosis, and augmentation in intracellular calcium concentration are, to a large extent, mediated by cAMP (cyclic adenosine monophosphate) and subsequent PKA (phosphorylase kinase A) activation [17]. Chronic effects, such as gene expression modulation, cellular growth enhancement, and antiapoptotic activity, can be ascribed to PI3K pathway, which overlaps with insulin/IGF-1 signaling. Further phosphorylation of aforementioned IRS by IR or IGF-1R introduces numerous binding sites for proteins bearing Shc (Src homology-2/α-collagen-related protein) homology domain, such as phosphoinositide 3-kinase (PI3-K). The latter can be also activated through GLP-1Rs. Triggered PI3K recruits AKT kinase (protein kinase B) by activation of CREB (cAMP response element-binding protein) protein. Consequent Bcl-2 (B-cell lymphoma 2) and Bcl-xl (B-cell lymphoma-extra-large) activation is able to induce protein synthesis and antiapoptotic actions through inhibition of glycogen synthase kinase 3β (GSK-3β), caspase-9, and Bcl-2-associated death promoter (BAD) [18,19,20,21]. Moreover, AKT action causes an inhibition of Forkhead box O (FoxO) and induces activation of the mammalian target of rapamycin (mTOR) [22]. The first molecule belongs to the transcriptional factor family involved in apoptosis induction and linked to apoptosis stimulating factors, such as Fas ligand (FasL) and Bcl-2-like protein 11 (Bim). The second was shown to be crucial to axonal regeneration [23]. Major cascade, through which GLP1 analogs can exert their function, is the mitogen associated protein kinase/extracellular signal regulated kinase pathway (MAPK/ERK). MAPK prevents apoptosis and limits oxidative stress and inflammatory response due to inhibition of caspase-9 and a nuclear factor kappa light-chain-enhancer of activated B cells (NF-κB). Upstream factors of this cascade, such as abovementioned IRS and Shc, compete for binding Grb2 (growth factor receptor-bound protein 2), and once bonded activate ERK, which subsequently translocates to the nucleus and phosphorylates transcriptional factors which take part in anabolic processes [15,24].

**Figure 1:**
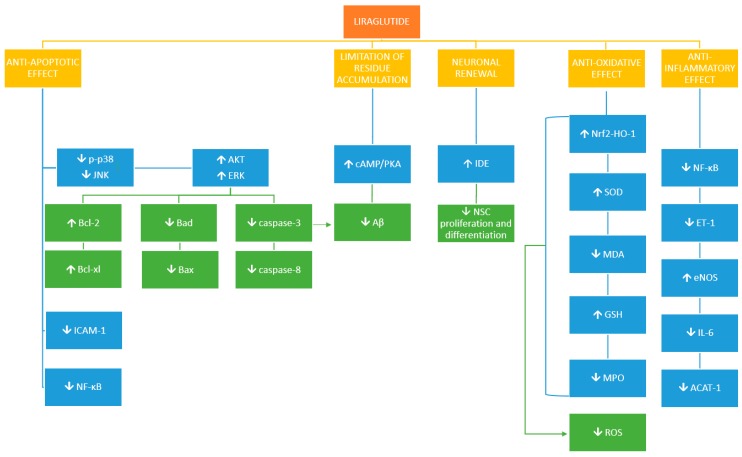
Proposed mechanisms of liraglutide activity; ↓ = reduction, ↑ = increase, p-p38 = phosphorylated p38, JNK-c = jun-NH2-terminal kinase, AKT = protein kinase B, ERK = extracellular signal-adjusted kinases, Bcl-2 = B-cell lymphoma 2, Bax = bcl-2-like protein 4, Bcl-xl = B-cell lymphoma-extra-large, Bad = Bcl-2-associated death promoter, NFκB = Nuclear Factor-kappaB, ICAM-1 = Intercellular Adhesion Molecule 1 Nrf2/HO-1-nuclear factor erythroid 2-related factor/heme oxygenase-1, MDA = malondialdehyde, GSH = glutathione, SOD = superoxide dismutase, MPO = myeloperoxidase, ROS = Reactive oxygen species, eNOS = endothelial nitric oxide synthase, ET-1 = Endothelin-1, eNOS = endothelial nitric oxide synthase, IL-6 = Interleukin-6, ACAT-1 = acyl-CoA: cholesterol acyltransferase 1, IDE = insulin degrading enzyme, Aβ = amyloid β, NSCs = neuronal stem cells, cAMP/PKA = cyclic adenosine monophosphate/phosphorylase kinase A.

**Figure 2:**
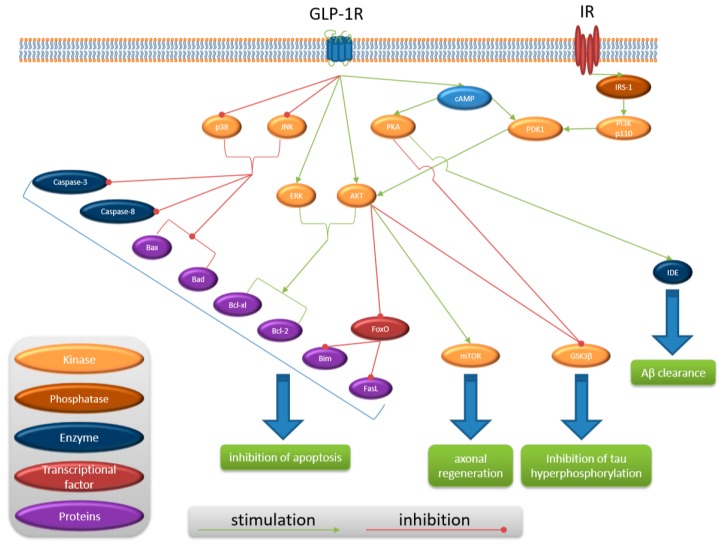
Proposed signaling transduction of liraglutide. GLP-1R = glucagon-like peptide-1 receptor, IR = insulin receptor, IRS-1 = Insulin receptor substrate 1, PI3K p110 = Phosphoinositide 3-kinase p110, PDK1 = Phosphoinositide-dependent kinase-1, JNK = c-jun-NH2-terminal kinase, AKT = protein kinase B, ERK = extracellular signal-adjusted kinases, Bcl-2 = B-cell lymphoma 2, Bax = bcl-2-like protein 4, Bcl-xl = B-cell lymphoma-extra-large, Bad = Bcl-2-associated death promoter, cAMP = cyclic adenosine monophosphate, PKA = phosphorylase kinase A, Bim = Bcl-2-like protein 11, FasL = Fas ligand or CD95L, FoxO = Forkhead box class O, mTOR = mammalian target of rapamycin, GSK3β = Glycogen synthase kinase 3 beta, IDE = Insulin-degrading enzyme, Aβ = amyloid beta.

Proposed mechanisms of liraglutide activity is presented in Figure 1 and proposed signaling transduction of liraglutide is presented in Figure 2.

## 2. Ischemia

During ischemic stroke hypoxia, an increased number of free radicals (appearing mostly in the reperfusion phase) can lead to an irreversible cascade of events resulting in cell death. Currently, the literature starts to suggest that liraglutide may reduce the effects of ischemic stroke by activating anti-apoptotic pathways, as well as limiting the harmful ascent of free radicals [25,26,27]. In the studies of Zhu et al. [28], liraglutide reduced the area of ischemia caused by MCAO (middle cerebral artery occlusion), limited neurological deficits, decreased hyperglycemia caused by stress, and presented anti-apoptotic effects by causing an increase in the expression of Bcl-2 and Bcl-xlproteins and reduction of Bax (bcl-2-like protein 4) and Bad protein production. These compounds regulate programmed cell death called apoptosis by either inducing (pro-apoptotic) or inhibiting (anti-apoptotic) certain molecular cascades. Secondly, the concentration of free radicals (ROS), quantified using DCFH-DA (dichlorofluorescein diacetate) method, has been significantly lowered. The results of research conducted by Brial et al. [25] are similar. Two week-long liraglutide pre-administration decreased activity of malondialdehyde (MDA), concurrently increasing superoxide dismutase level (SOD) and glutathione (GSH) subsequent to MCAO induction. As a result of TUNEL method analysis (Terminal deoxynucleotidyl transferase dUTP nick end labeling), significant diminishment in TUNEL-positive cells number—that is, ones which underwent apoptosis—has been observed. TUNEL is a method for detecting apoptotic DNA fragmentation, widely used to identify and quantify apoptotic cells, or to detect excessive DNA breakage in individual cells [29]. The results demonstrate that GLP-1 agonist liraglutide may attenuate the neuronal damage following cerebral ischemia in rats by preventing apoptosis and decreasing oxidative stress. Dong et al. [30] have made an effort to assess the impact of liraglutide application one day after the ischemic event (MCAO). The group of rats treated at doses of 100 and 200 μg/kg obtained favorable results in mNSS (modified neurological severity score), which is a behavioral test used to assess the severity of stroke symptoms in rodents [31].

An accumulation of 18F-FDG (18F-fluorodeoxyglucose) has been observed, indicating increased glucose metabolism after liraglutide administration, which may suggest return of neuronal function [30]. PET (positron-emission tomography) with 18F-fluorodeoxyglucose (18F-FDG) is able to detect subtle changes of glucose metabolism in rats following ischemic stroke. The elevation of marker concentrations, such as NeuN (neuronal marker), GFAP (Glial fibrillary acidic protein, glial cell marker), and vWF (von Willebrand Factor, marker of endothelial cells) following liraglutide treatment suggest that it may affect the neurovascular unit (i.e., unit consisting of neurons, glial cells, and vascular cells in combination with extracellular matrix), causing its remodeling [30]. The reconstruction of this unit seems to be crucial for recovery after stroke [32,33,34]. In Li et al.’s study on mice after MCAO [35], liraglutide managed to decrease concentrations of proapoptotic factors, such as NF-κB, ICAM-1 (Intercellular Adhesion Molecule 1), caspase-3, and TUNEL-positive cells, in addition to showing anti-radical properties. Moreover, it significantly decreased Bax expression and the Bax/Bcl-2 ratio, leading to reduced apoptosis, possibly through stimulation of AKT and eNOS (endothelial-NOS) signaling. AKT pathway activation in ischemic regions has been reported to reduce ischemia- and reperfusion-induced damage by modulating the endothelial nitric oxide pathway. Observations from studies in human umbilical vein endothelial cells (HUVECs) are consistent [36].

Liraglutide was reported to normalize TNFα-induced (tumor necrosis factor alpha) pro-oxidant levels. The effect may result from decreased expression of the NADPH oxidase subunit, reduced membrane translocation of PKC-α (Protein kinase C alpha), and overexpression of SOD and catalase. The researchers assumed that there may be a correlation with TNF-α -induced activation of NF-kB. Studies by Deng et al. [37] of diabetic rats with induced MCAO brought results showing the activation of the Nrf2/HO-1 signaling pathway (nuclear factor erythroid 2-related factor/heme oxygenase-1) by liraglutide. Consistently, the reduction of neurological deficits and the MPO (myeloperoxidase) activity has been observed. The Nrf2/HO-1 pathway activates and regulates antioxidant enzymes and is associated, among others, with the response of nerve and glial cells and endothelium to cerebral ischemia [38]. Moreover, researchers associate activation of the Nrf2 pathway with protective effects on limitation of damage caused by reperfusion [39,40]. Zhu et al. [29] suggested that the anti-apoptotic effect of liraglutide may be linked to an impact of GLP-1 receptor activation on intracellular pathways associated with apoptosis, including PI3K/AKT and MAPK. Furthermore, liraglutide reduced the expression of phosphorylated p38 and JNK (c-jun-NH2-terminal kinase), but increased the expression of AKT and ERK (extracellular signal-adjusted kinases). Previous studies have shown that the activation of PI3K/AKT and ERK can effectively inhibit ROS generation by regulating the expression of the Bcl-2 family [41]. As important anti-oxidative proteins, Bcl-2 and Bcl-xl efficiently scavenge free radicals and inhibit the formation of superoxide, which consequently alleviates the oxidative damage caused by ROS overexpression in ischemic neurons [42,43]. Meanwhile, Bcl-2 has strong antioxidant activity, and PI3K/AKT and ERK can up-regulate the expression level of Bcl-2 to inhibit ROS generation in ischemic damage [44,45]. Interestingly, ROS inhibits AKT and ERK phosphorylation and promotes cell apoptosis in turn [46,47,48]. The authors stated that activation of PI3K/AKT pathway and ERK resulted from GLP-1R triggering and caused consequent moderation of apoptosis regulator concentration. Reduced JNK expression and phosphorylated p38 decreased the intensity of apoptosis through reduction in caspase-8 and -3 activation. Other researchers also observed a similar effect of liraglutide consisting in the activation of the PI3K/AKT pathway [49], ERK [50], as well as inhibition of JNK [51] and phosphorylated p38 [52].

## 3. CNS Inflammation and Atherosclerosis

There are reports on possible relevance of liraglutide to slowing down the development of atherosclerotic disease, which is one of the major causes of cerebral ischemic events [53]. Strokes caused by atherosclerotic lesions in carotid, vertebral, and brain arteries result from critical stenosis of these vessels causing restriction of cerebral blood flow and lesion-connected thrombosis. According to Chung et al. [54], large-artery atherosclerosis was the most common subtype for anterior cerebral, middle cerebral, vertebral, and anterior and posterior inferior cerebellar artery territory infarctions.

In the study of Gaspari et al. [55], researchers obtained results demonstrating liraglutide’s efficacy in stabilization of atherosclerotic plaque, as well as in counteracting early formation and its further development. Both of these effects were partially dependent on the GLP-1 receptor. According to the results of Dai et al. [56], the probable mechanism of liraglutide’s action is related to the inhibition of NF-κB phosphorylation. The process leads to the reduction of ET-1 (Endothelin-1) expression, which is a strong vasoconstrictor [57,58], and increase the expression of eNOS (Endothelial nitric oxide synthase), a vasodilator. Elevated production of vasoconstrictive factors and consequently reduced vasodilatation are known factors contributing to endothelial dysfunction and atherogenesis [57]. Nonetheless, the basis of atherosclerosis pathology is chronic endothelial inflammation [59,60,61,62]. Liraglutide was shown to be effective in limiting the production of IL-6 (Interleukin-6), a potent pro-inflammatory cytokine, by acting on a PMA cell (phorbol 12-myristate 13-acetate), which activates NF-κB. The cells exposed to PMA presented increased production of IL-6, however, this action was reversed by administration of liraglutide [56].

Furthermore, Tashiro et al. [63], in human macrophages and apoE −/− mice studies, suggest that liraglutide causes suppression of oxidized LDL-induced foam cell formation. Administration of liraglutide to mice prevented the development of atherosclerotic lesions induced by macrophages. They associate these actions with the down-regulation of ACAT-1 (acyl-CoA: cholesterol acyltransferase 1), an enzyme found in macrophages responsible for the conversion of free cholesterol to CE (cholesterol ester), which are stored in macrophages. Interestingly, the results of a study conducted by Xin et al. [64] suggest that ACAT-1 regulation may be performed with the involvement of previously mentioned signaling pathways, such as ERK, p38MAPK, and JNK.

## 4. Alzheimer’s Disease

Epidemiological evidence suggests a close connection between type 2 diabetes (DM2) and Alzheimer’s disease (AD) [65,66]. A decrease in insulin sensitivity in CNS, as a result of persistent elevation of cerebral glucose concentration, causes neuronal cell death in numerous interconnected mechanisms, including oxidative stress, mitochondrial dysfunction [67], and neuroinflammation [68,69] that are observed in these disorders [70]. Both conditions share several molecular pathways which lead to the cell degeneration [71]. In histopathological examination of Alzheimer’s disease, there are extra- and intracellular residues called plaques—built from *β*-amyloid and neurofibrillary tangles (NFTs)—which are aggregates of tau protein, which binds to microtubules [70]. The process of protein accumulation induces progressive loss of neurons [72]. The search for effective treatment is still on-going, and undeniable disappointment with current solutions impels scientists to explore new perspectives. Studies have shown that GLP-1 and GLP-1 analogs are able to induce cell proliferation in vivo and in vitro [73,74]. Adequate receptors have been found both in the brains of humans and rodents [75,76]. GLP-1R expression has been reported in the cerebral cortex, especially occipital and frontal lobes, the hypothalamus, and the thalamus. What is more, they have been found within such cerebral areas as the pyramidal layer of the hippocampus, the granule layer of the dentate gyrus, and Purkinje cells of the cerebellum. In glial cells, GLP-1Rs can be localized only in pathological conditions [77,78,79]. It is worth noting that the phenotype of GLP-1 receptor-deficient mice is connected to learning deficits and enhanced seizure severity, which is reversible after hippocampal transfer of the lacking gene. Moreover, the overexpression of the above-mentioned gene is correlated with improved cognition [80]. There are hopes that endogenous stem cell stimulation leading to new neuron proliferation and differentiation may provide a new way to fight neurodegenerative diseases. What is interesting is that dietary energy restrictions have been observed to prevent neuronal degeneration [81]. In experimental AD mice models, caloric restriction has proven to reduce Aβ (amyloid beta) and NFT loads. Clinical studies have shown that administration of GLP1 analogs decreases food intake, hunger, and body weight [82,83]. The impact of GLP-1 signaling on energy availability and glucose metabolism may contribute to preserving cognitive functions. Nevertheless, the above-mentioned actions of GLP-1R activation are being considered as directly mediated by BDNF (Brain-derived neurotrophic factor), rather than secondary to limited food intake [84,85]. The role of BDNF in learning and memory has been established by investigations on in vivo rodent models [86], and perspectives of BDNF targeting in CNS disorders has been reviewed by Pezet and Malcangio [87].

Parthsarathy and Hölscher have analyzed duration-dependent differences in effectivity of treatment with liraglutide. They used APP/PS1 transgenic mice, which are characterized by early plaque deposition, which have been previously linked to insulin signaling deficiency [88,89]. Ki67, BrdU (pyrimidine analogue of thymidine), and DCX (double-cortin) staining have been utilized to assess proliferation and differentiation levels in mice treated with liraglutide through the extending period of time. DCX is a microtubule associated protein expressed almost exclusively in immature neurons. Both BrdU and Ki-67 protein (also known as MKI67) are cellular markers for proliferation [7]. During the study, responses to 7-day and 37-day long treatment liraglutide (25 nmol/kg of body weight) were compared. Presented data of theirs suggest that acute administration is sufficient to trigger trophic reaction of neuroblasts but chronic administration is necessary to induce a differentiation process and obtain mature neurons [90]. Liraglutide did not appear to affect the differentiation into glial tissue. There is evidence that GLP-1 signaling is implicated in ciliary neurotrophic factor (CNTF)-dependent cell proliferation. CNTF in vivo administration has been correlated with increased expression of GLP-1 immunoreactivity in the hypothalamus. Moreover, hypothalamic cell cultures derived from mice with inoperative GLP-1R genes did not demonstrate CNTF- induced BrdU incorporation. The facts may imply functional requirement of GLP-1R activation [91]. A recent study by Sun et al. [92] implies that imbalance between GABAergic (Gamma-Aminobutyric Acid) and glutamatergic neurotransmission may contribute to impaired neurogenesis in AD, which may impair some aspects of learning and memory [93,94]. Liraglutide has been previously proven to affect GABAergic and glutamatergic neurotransmission [95,96,97]. The mechanism of the regulation remains unclear, but it is speculated that the action is mediated through GLP-1R. In a study by Gilman et al. [97], GLP1 pre-treated neurons showed attenuated calcium responses to glutamate and membrane depolarization. Therefore, it can be deduced that GLP-1R activation may have an influence on neuroprotection against glutamate-induced cell death. In two following studies, McClean et al. (2011, 2013) assessed whether liraglutide possess neuroprotective properties [98,99]. Two-month long treatment of 7 and 14-month old APP/PS1 mice indicated that liraglutide (25 nmol/kg of body weight) is able to improve spatial memory, significantly reduce pathological inclusion count, and inflammation response by 30–50%. (Table 1) What is more, some renewal properties have been observed. Compared to saline-treated subjects, there was an augmentation in neuronal progenitor cell number reaching 50% within the hippocampal area known as dentate gyrus. The above-mentioned effects might occur due to an increase in the insulin degrading enzyme, which was present in studied mice.

Aβ is a product of the APP (Amyloid precursor protein) cleavage. Potential pathways of Aβ processing can be generally divided into non-amyloidogenic and amyloidogenic. In the latter, β and γ secretases cleave the APP to 40 and 42 amino acid long peptides prone to forming toxic species of Aβ [104,105]. There are several potential mechanisms through which Aβ can be cleared from neuronal extracellular space [104,106]. Probably the most relevant is via transcytosis across the blood barrier into the vascular lumen [107]. The second consists of proteolytic degradation of Aβ by enzymes such as IDE. The enzyme is secreted from microglial cells and neurons, and degrades Aβ extracellularly and on the cell surface. The third way is to potentiate more stable fibril formation, which is thought to be less toxic than soluble oligomers [108,109,110]. Lastly, it is known that the activation of an innate immune system, especially microglia and the complement signaling pathway, can be caused by the presence of the aggregates. While the acute immune response may initially create a positive impact by increasing misfolded protein clearance, the chronic activation of inflammatory pathways may lead to neuronal damage [111,112]. Liraglutide, through its binding to GLP-1R and consequent activation of PI3K/MAPK dependent pathways, may have a positive impact on Aβ trafficking and clearance by increasing the presence of Aβ transporters in cerebrospinal fluid [113,114,115,116]. What is more, studies suggest that the cAMP/PKA signaling pathway is able to alter IDE expression in a mixed model of DM2 and AD [110,117]. Although the exact mechanism underlying the linkage of IDE with DM2 and AD has not been fully elucidated, it can be hypothesized that GLP-1R may regulate IDE expression by modulating the cAMP/PKA signaling pathway and affect Aβ proteolytic degradation. Liraglutide caused a delay in progression of neurobehavioral and neuropathological changes, which has also been reported by Hansen et al. (2015) in SAMP8 mice. Interestingly, in this model, administration of 100 or 500 g/kg/day of s.c. for four months appeared to be most effective in the lower dosage [101]. Similar results were obtained by Holubová et al. using liraglutide; they achieved significant reduction in overall number of amyloid plaques, especially in the CA1 (cornu ammonis 1) area [103], which is believed to be one of the particularly vulnerable regions in AD [118]. Pathological overexpression of caspase-3 in APP/PS1 mice has been limited due to chronic administration of the drug. The above-mentioned protein plays several roles in AD pathogenesis. involving amyloidosis [119], NFT formation [120], and neuronal apoptosis [121]. Inhibition of caspase-3 activity may prove to be useful in AD treatment in the future. Batista et al. (2018) stated that liraglutide’s protective action is correlated with cAMP/PKA activation. The results imply that injections of amyloid β oligomers (AβOs), used to induce a memory-deficit, caused a decrease in PKA activity in the mouse hippocampi [100]. PKA activity has been previously described as decreased in AD brain [122], and Aβ was found to be responsible for the impairment of enzyme action [123]. A 1-week long pre-treatment (dose) was able to limit the unbeneficial effects of AβOs (amyloid beta oligomers) in PKA activity. What is more, in this study, liraglutide was shown to be able to attenuate AβOs binding to synapses, suggesting another potential mechanism of its action. In contrast, PKA activation may have a different impact regarding taupathy. Van der Harg and Bangel et al. [124] postulated that PKA activation is possibly able to increase tau phosphorylation and subsequent NFT accumulation. The researchers observed elevated PKA activity after streptozocin-induced hipoinsulinemia. Interestingly, changes in activity of the kinase were reversible upon insulin treatment. Therefore, it can be presumed that this convertible reaction may result from its adaptive character as a physiological response to stress. On the other hand [125], in the study of Myeku et al., stimulation of PKA activity correlated with a decrease in overall tau aggregation in mice with the early stage of tau pathology. The authors related the action to proteasome activity increased by PKA-mediated phosphorylation. Liraglutide seems to affect tau pathology. Hansen et al. (2016) [102] reported favorable changes in motor function, including reduction in neurological symptoms, such as hind limb clasping specific to the transgenic hTauP301L mouse model. The GLP-1 agonist lessened the clasping rate to 39%, fully prevented lethality resulting from clasping, and diminished neuronal phospho-tau load by 60–70% at both the pontine and medullary level. Previous studies have shown that hyperphosphorylation of tau results from activation of several cell signaling pathways and metabolic abnormalities, including down regulation of protein phosphatases, and upregulation of GSK-3β [126]. Researchers suggest that liraglutide increased brain insulin signalling activity, leading to inhibition of GSK-3β through AKT-increased phosphorylation, and reversed hyperphosphorylation of tau protein. A 4-week long treatment resulted in a time-dependent recovery of phosphorylation of both AKT and GSK-3β and ameliorated tau hyperphosphorylation [127]. Similar results were obtained by Ma et al. [128] in their db/db mice study. Liraglutide diminished age-dependent increase of tau phosphorylation within the hippocampus and prevented the dysregulation of AKT and GSK-3β phosphorylation, which occurs in db/db mice with age. Interestingly, insulin administration did not show a similar protective effect, suggesting a mechanism independent of the insulin signaling pathway. Qi et al. [129] stated that liraglutide may increase the expression of GLP-1R in the hippocampus, improve cognitive function of mice with AD, and reduce Aβ-stimulated tau hyperphosphorylation, attributed to AKT activation and subsequent GSK-3β inhibition. What is more, Batista et al. [100] reported that liraglutide reduces tau hyperphosphorylation caused by Aβ in non-human primates (NHPs). Taken together, these results suggest a beneficial effect of insulin and GLP-1 signaling on tau aggregation.

## 5. Conclusions

The information presented above allows for considering liraglutide as a promising drug in the treatment of conditions other than type 2 diabetes. If GLP-1 analogs are demonstrated to have clinically meaningful anti-sclerotic activity in humans, one potential application may be to reduce the burden of certain neurodegenerative disorders. Liraglutide seems to promote neuronal survival and attenuate apoptosis and oxidative stress in the brain. Both the reduction of oxidative stress and anti-apoptotic effects appear to play a role in neurological recovery following cerebral ischemia. The moderation of free radical creation and aggregation of β-amyloid may prove to be helpful in reducing neurodegeneration connected with AD. Potential properties stimulating neuronal renewal, if proven, would find application in the treatment of various forms of dementia. Studies assessing neuroprotective properties of liraglutide need to be strengthened in order to plausibly evaluate its usability.

## Figures and Tables

**Table 1:** Summary of reviewed results.

Authors	Subject of Study	Dose of Liraglutide	Results
**Batista et al. (2018) [100]**	Non-human primate model, male Swiss mice	0.006 mg/kg for the first week and 0.012mg/kg thereafter 25 nmol/kg/day for 7 days	↑ cAMP/PKA ↓ Aβ plaques ↓ memory impairment ↓ synapse loss
**Briyal et al. (2014) [25]**	Sprague-Dawley rats	Pretreated 50 μg/kg per day for 14 days	↓ infract size after MCAO, ↓ neurological deficit, ↑ Bcl-2, ↓ Bax, ↓MDA, ↑ GSH, ↓ TUNEL-positive cells, ↑ SOD
**Dai et al. (2013) [53]**	HUVECs	10, 100, 1000 ng/mL for 6–24 h	↓ NF-κB, ↓ ET-1, ↑ eNOS, ↓ IL-6
**Deng et al. (2018) [37]**	Sprague-Dawley rats	100 μg/kg twice daily for 7 days prior MCAO	↓ infract size after MCAO, ↓ neurological deficit, ↑ SOD, ↓ MPO
**Dong et al. (2017) [30]**	Sprague-Dawley rats	1 day after MCAO–50, 100, 200 μg/kg per day for 4 weeks	↑ mNSS, ↑ 18F-FDG, ↑ NeuN, ↑ GFAP, ↑ vWF
**Hansen et al. (2015) [101]**	SAMP8 mice	100 or 500 g/kg/day s.c. for 4 months	↑ memory retention; ↑ CA1 neuron number
**Hansen et al. (2016) [102]**	hTauP301L transgenic mice	500 mg/kg/day for 6 months	↓ NFTs ↑motor function
**Holubová et al. (2019) [103]**	APP/PS1 mice	0.2 mg/kg /day for 3 months	↓ Aβ plaques ↓ caspase-3
**McClean et al. (2011) [98]**	APP/PS1 mice	25 nmol/kg for 2 months	↓ synapse loss ↓ Aβ plaques ↓ memory impairment ↑ recognition test score
**McClean et al. (2013) [99]**	APP/PS1 mice	25 nmol/kg for 2 months	↓ Aβ plaques ↑ neuronal progenitor cell count ↓ inflammatory response in CNS
**Li et al. (2016) [35]**	db/db mouse	0.1 mg/mL administered intraperitoneally during the 0, 3, 6, or 12 h reperfusion periods following MCAO	↓ ROS, ↓ NF-κB, ↓ ICAM-1, ↓ caspase-3, ↓ TUNEL-positive cells ↑ p-AKT, ↑ p-eNOS
**Parthsarathy et Hölscher (2013) [90]**	APP/PS1 transgenic mice	25 nmol/kg per day for 7 days 25 nmol/kg per day for 37 days	↑NSC proliferation ↑NSC differentiation
**Shiraki et al. (2012) [36]**	HUVECs	Pre-incubated 30nM/mL for 1 h	↓ ROS, ↑ SOD, ↑ catalase
**Tashiro et al. (2014) [63]**	Human macrophages and apoE−/− mice	107 nmol/kg/day for 4 weeks	↓ foam cells, ↓ macrophage-driven atherosclerotic lesions,
**Zhu et al. (2016) [28]**	Sprague-Dawley rats	1 h after MCAO–100 μg/kg per day for 1, 3 and 7 days	↓ infract size after MCAO, ↓ neurological deficit, ↑ Bcl-2, ↑ Bcl-xl, ↓ Bax, ↓ Bad, ↓ ROS

Note: ↓ = reduction, ↑ = increase, MCAO = middle cerebral artery occlusion, Bcl-2 = B-cell lymphoma 2, Bax = bcl-2-like protein 4, MDA = malondialdehyde, GSH = glutathione, TUNEL = Terminal deoxynucleotidyl transferase dUTP nick end labeling TUNEL-positive cells-apoptotic cells, SOD = superoxide dismutase, Bcl-xl = B-cell lymphoma-extra-large, Bad = Bcl-2-associated death promoter, ROS = Reactive oxygen species, mNSS = modified neurological severity score, 18F-FDG = 18F-fluorodeoxyglucose, NeuN = neurons marker, GFAP = Glial fibrillary acidic protein, vWF = von Willebrand Factor, s.c. = subcutaneous, NFκB = Nuclear Factor-kappaB, ICAM-1 = Intercellular Adhesion Molecule 1, p-AKT = phosphorylated protein kinase B, p-eNOS = phosphorylated endothelial nitric oxide synthase, HUVECs = human umbilical vein endothelial cells, MPO = myeloperoxidase, ET-1 = Endothelin-1, eNOS = endothelial nitric oxide synthase, IL-6 = Interleukin-6, NFTs = neurofibrillary tangles, Aβ = amyloid β, db/db mouse = mouse with leptin receptor db mutation, NSCs = neuronal stem cells, CA1 = cornu ammonis, cAMP/PKA = cyclic adenosine monophosphate/phosphorylase kinase A.
